# Simultaneous Electrochemical Detection of Pb and Cd by Carbon Paste Electrodes Modified by Activated Clay

**DOI:** 10.1155/2022/6900839

**Published:** 2022-01-19

**Authors:** Niraka Blaise, Hambate Gomdje Valéry, Raja Maallah, Mohamed Oubaouz, Bakary Tigana Djonse Justin, Edwin Andrew Ofudje, Abdelilah Chtaini

**Affiliations:** ^1^National Advanced School of Engineering, University of Maroua, P.O. Box 46, Maroua, Cameroon; ^2^Team of Molecular Electrochemistry and Inorganic Materials, Faculty of Sciences and Technology, Sultan Moulay Slimane University of Beni Mellal, Beni Mellal, Morocco; ^3^Department of Chemical Sciences, Mountain Top University, Prayer City, Ogun State, Nigeria

## Abstract

Calcinated and acidified clay modified carbon graphite electrode was deployed in the simultaneous evaluation of traces of Pb^2+^ and Cd^2+^ in solution. After 5 minutes of accumulation in the circuit, the sensitivity of the electrode was evaluated in a solution of Na_2_SO_4_ (0.1 M) by square wave voltammetry on the one hand with Pb (II) and on the other hand with Cd (II). Several experimental conditions such as the composition of the carbon clay paste, the effect of preconcentration time, the sweeping speed, concentration effect, media pH, and interference ionic response to the electrochemical response of the working electrode were examined. It was observed that, after 5 minutes of preconcentration, detection limits of 0.15513 *μ*mol·L^−1^ and 0.24227 *μ*mol·L^−1^ were obtained for Pb^2+^ and Cd^2+^ in the electrolyte solution and 0.08438 *μ*mol·L^−1^ and 0.46522 *μ*mol·L^−1^, respectively, when tap water was used. The detection was effective by square wave voltammetry with a more intense current density with respect to lead.

## 1. Introduction

Heavy metals are used in many areas of life, but excessive and uncontrolled use can be harmful to the environment and to human health [1]. The presence of these heavy metals in aquatic environments results from natural and anthropogenic activities [2]. Excessive and uncontrolled use of heavy metals can increase their concentration even in drinking water and could be harmful to living organisms. Prolonged exposure even to low doses of lead (Pb^2+^) and cadmium (Cd^2+^) ions by inhalation or ingestion can cause acute and chronic nuisances such as decrease in hemoglobin level, headache, nausea, dizziness, vomiting, constipation or diarrhea, breathing difficulties, haematuria, gastrointestinal haemorrhage, acute hepatic failure, cardiovascular disorder, neurotoxic effect (in children), abdominal cramps, and impact on the immune system [3, 4].

This requires increased and real-time monitoring with a view to either reducing their presence in drinking water or complete removal. Several techniques for analyzing traces of heavy metals in water have been used, among others which are IC (ion chromatography), ICP-AES (atomic emission spectroscopy coupled with inductive plasma), AAS (atomic absorption spectroscopy), and ICP-MS (mass spectroscopy coupled with an inductive plasma) [5–7]. These techniques certainly give convincing results, but AAS, for example, is a destructive method, limited just to chemical elements in the atomic state; in addition, the element to be analyzed must be known in advance, and its concentration must be at the trace scale at the risk of damaging the machine [8]. In addition, ICP-MS and ICP-AES have the drawback of pretreating the samples before analysis and with very expensive equipment maintenance cost [9, 10]. Under another prism, the rapid reaction, the great sensitivity, and the portability relating to electrochemical methods gives them a remarkable attraction [11, 12]. To this end, for several decades, the use and improvement of the sensitivity of electrochemical sensors to both organic pollutants and heavy metals has been of growing interest for electrochemists [13]. Because of their adsorbing power and their good cation exchange capacity, smectite-type clays have been used either as a carbon electrode dopant [14] or as a constituent of organic-inorganic hybrid materials [15, 16] in view of modifying carbon paste electrodes.

As part of this work, an electrochemical sensor based on carbon graphite paste (CPE) was functionalized by acidified and calcinated clay (AAC) for the electroanalysis of Pb^2+^ and Cd^2+^ in solution by cyclic voltammetry and square wave voltammetry. The search for optimum parameters, such as the best CPE/AAC (mass for mass) ratio, the best preconcentration time, the best scanning speed, the ideal pH value of the solution, and the ionic concentration giving the best response in current run, was made. In addition, the interference effect and the performance and stability of the modified CPE-AAC electrode were also investigated.

## 2. Materials and Methods

### 2.1. Materials and Chemicals

The clay material used in this work was the subject of previous work [17]. The reagents used in this work are analytically reliable, therefore have not undergone any prior purification. The binder used (paraffin oil) and the carbon graphite powders were supplied by the firm Sigma-Aldrich. Cadmium nitrate tetrahydrate and lead nitrate were provided by Riedel-de Haen. Finally, the extrapure sodium sulfate used comes from Scharlau Chemie.

### 2.2. Devices

A potentiostat (PGSTAT 100, Eco Chemie BV, Utrecht) connected both to a computer equipped with Volta lab master 4 software (for data processing) and to an electrochemical cell equipped with a saturated calomel reference electrode (SCE), an auxiliary platinum electrode, and a working electrode made of the carbon/calcinated and acidified clay composite (CPE-ACC) electrochemical analyzes were deployed in the measurement. The pH control of the reaction medium was possible using a HANNA pH meter (HI 2210).

### 2.3. Preparation of Modified Electrodes

In fair proportions, a homogeneous mixture of clay and graphite carbon was prepared. Paraffin oil was added to the powder so as to obtain a homogeneous paste. Ultimately, ethanol was added to the paste as an inert and volatile solvent. The paste obtained was used to fill the cylindrical cavity of a tube (acting as a working electrode) whose orifice has a surface area of 0.1256 cm^2^. The electrical supply of the dough is ensured via a carbon bar.

### 2.4. Procedure and Measurement

The electrochemical detection of lead (II) and cadmium (II) was carried out by immersing the working electrode (CPE-ACC) in a solution of Na_2_SO_4_ (0.1 M) containing on the one hand 0.242 × 10^−6^ *μ*mol·L^−1^ of lead (II) and on the other hand 0.259 × 10^−6^ *μ*mol·L^−1^ of cadmium (II). After 5 minutes of accumulation in open circuit and at pH = 7, the electrochemical measurements were made by SWV in the potential range −1.5 V to 1.5 V, according to previous work [18].

### 2.5. Characterization Techniques

The SEM micrographs of the CPE/ACC were taken in Hitachi (Japan) S-3000H electron microscope with an accelerating voltage of 15 kV.

## 3. Results and Discussion

### 3.1. Effect of CPE/AAC Mass Ratio in Electrolytic Medium

**Figure 1:**
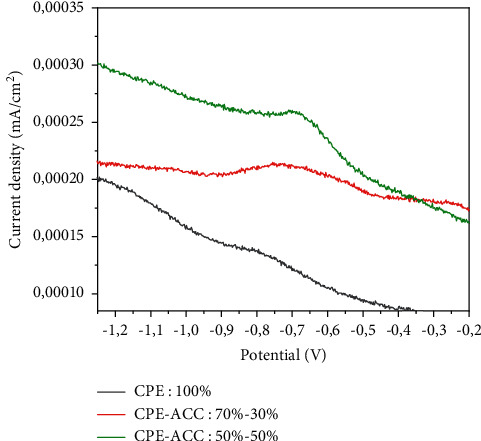
CPE-ACC SWV responses at different proportions (Hg/Hg_2_Cl_2_).

Figure 1 shows the responses of the electrodes in a solution of Na_2_SO_4_ (0.1 M) in the presence of lead (II) ions (2.42 *μ*M). The study was carried out by SWV in the potential range [−1.5 V; +1V]. The voltammograms show peak currents around −0.7 V, reflecting the detection of lead (II) ions in the solution. These peak currents increase as the electrode is enriched with clay. Following the various responses, the 50%-50% electrode was chosen for the rest of our study.

### 3.2. CPE-ACC Characterization by Scanning Electron Microscopy

**Figure 2:**
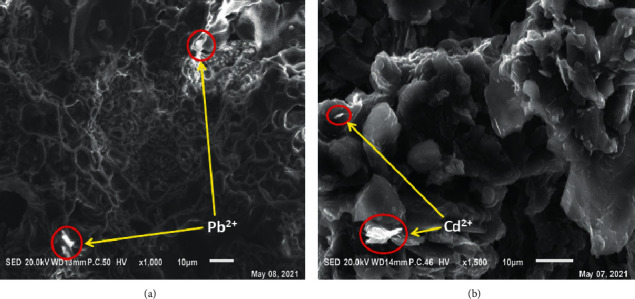
SEM of the CPE-ACC: (a) with Pb and (b) with Cd.

Figure 2 shows the scanning electron microscopy images of clay-modified carbon paste in the presence of Pb (II) and Cd (II). Scanning electron microscopy of the electrodes after use shows the predominance of the interfollial microstructure of the clays constituting the electrode. In addition, graphitic structure of the carbon paste is also observable. These images show an agglomerate probably due to the formation of microcrystalline complexes.

### 3.3. Sensitivity of CPE and CPE-ACC Electrodes

#### 3.3.1. Behavior of CPE-ACC with Pb

**Figure 3:**
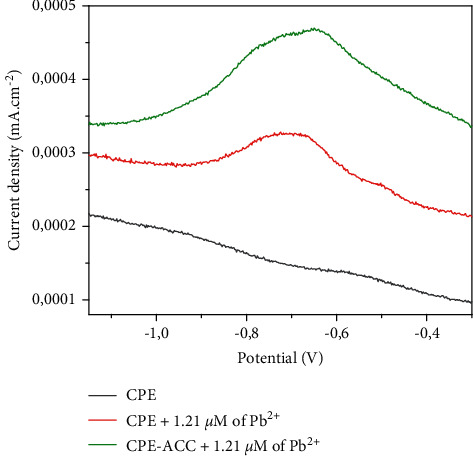
Sensibility of electrodes with Pb^2+^ by SWV.

Figure 3 shows the square wave voltammograms of the CPE and CPE-ACC electrodes. These voltammograms were recorded in a 0.1 M of Na_2_SO_4_, in the potential range −1.5 V and 0.5 V. After 5 minutes of preconcentration in an open circuit, the peak currents around −0.7 V on the CPE and CPE-ACC electrodes reveal the presence of lead in the reaction medium. The increase in peak currents from 0.3253 mA·cm^−2^ (with CPE) to 0.4681 mA·cm^−2^ (with CPE-ACC) not only shows that the CPE-type electrode has indeed been modified but also that the modification boosts the recognition of lead. Previous work has shown similar behavior [19, 20].

#### 3.3.2. Behavior of CPE-ACC with Cd

**Figure 4:**
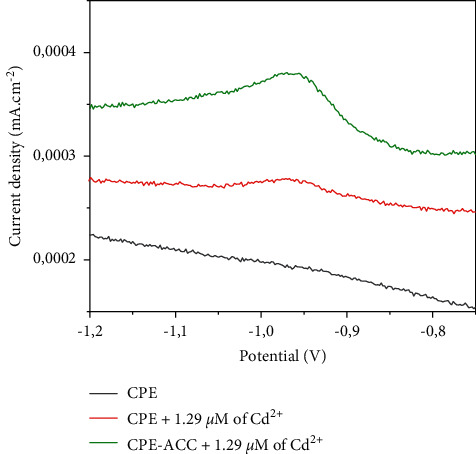
Sensibility of electrodes with Cd^2+^ by SWV.

By SWV, the electrochemical behavior of CPE-ACC was studied as illustrated in Figure 4. Cyclic voltammograms were recorded in 0.1 M of Na_2_SO_4_, in the potential range of −1.5 V to 1 V. After 5 minutes of preconcentration, the voltammograms show peak currents around −0.95 V indicating the presence of cadmium. These relatively low peak currents compared to those obtained in the presence of lead which can be explained by low energy interactions (which may suggest physisorption) between cadmium and the CPE-ACC composite [21]. The fluctuation in the positive direction of peak currents from 0.2775 mA/cm^2^ to 0.3794 mA/cm^2^ attests that the modification of CPE by the Makabaye ACC brings added value to the recognition of cadmium in solution. By comparing the peak currents of the CPE-ACC electrode against the metal ions Pb^2+^ and Cd^2+^ under the conditions of the experiment, it is clear that this electrode is more reactive and sensitive towards lead. Similar results have been reported by previous work [22].

### 3.4. Optimization of Some Parameters

#### 3.4.1. Study of the Effect of Ionic Concentration

**Figure 5:**
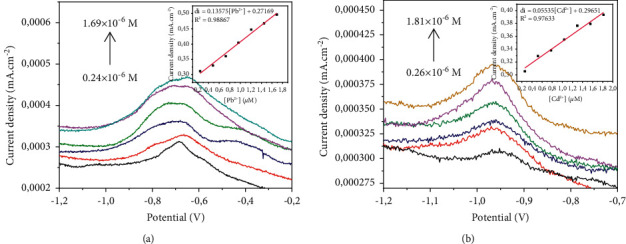
Effect of concentration (Hg/Hg_2_Cl_2_; pH = 7; 0,1 M of Na_2_SO_4_). (a) Pb^2+^. (b) Cd^2+^.

In order to appreciate the effect of ionic strength on the electrochemical behavior of the proposed working electrode, electrochemical measurements were made with both lead (II) and cadmium (II). The concentrations of the metal ions in solution were therefore varied as shown in Figures 5(a) and 5(b). According to these figures, the observation is that the peak currents increase linearly with the concentration of metal ions in solution with correlation coefficients of the order of 0.989 and 0.977, respectively, for lead (II) and cadmium (II). This increase is more pronounced with Pb^2+^ (Figure 5(a)) than with Cd^2+^ (Figure 5(b)). This reiterates the fact that the CPE-ACC electrode is more reactive to Pb^2+^ than to Cd^2+^. Besides, the detection limits were 0.15513 *μ*M and 0.24227 *μ*M, respectively, for Pb^2+^ and Cd^2+^.

#### 3.4.2. Study of the Effect of Scan Rate

**Figure 6:**
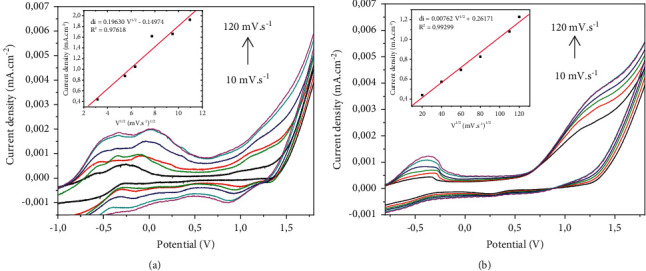
Scan rate effect (Hg/Hg_2_Cl_2_; pH = 7; 0.1 M of Na_2_SO_4_). (a) Pb^2+^. (b) Cd^2+^.

By varying the scanning speed from 10 mV/s to 120 mV/s in the reaction medium, the extent of the electrode area involved in the electrochemical analysis was evaluated. Figures 6(a) and 6(b) illustrate the behavior of the electrode. The linear increase of the anode peak currents with the square ratio of the scanning speed (correlation coefficient equal to 0.976 and 0.939, respectively, with Pb^2+^ and Cd^2+^) shows that there is a diffusion of the analyte on the surface of the electrode [19, 23].

#### 3.4.3. Study of the Effect of Accumulation Time

**Figure 7:**
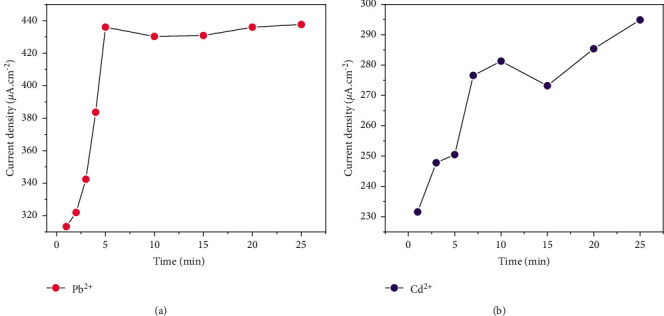
Effect of accumulation time by SWV (Hg/Hg_2_Cl_2_; pH = 7; 0.1 M of Na_2_SO_4_).

In the time span between 1 and 25 minutes, the accumulation of metal ions on the surface of the working electrode was studied by SWV. Figures 7(a) and 7(b) are illustrations thereof. With reference to Figure 7(a), the current density increases rapidly as time increases up to five minutes. This reflects a rapid build-up of lead on the electrode surface because the adsorption sites are readily available. Beyond 5 minutes, the current density values form a bearing. A saturation of the surface of the working electrode by the analyte could justify this behavior. Figure 7(b) shows a continuous increase in current density over time. This observation can also be justified by the fact that the Cd^2+^ ions are increasingly adsorbed during the study time. Previous work reveals similar behavior of heavy metals on a modified carbon graphite electrode [12]. The presence of cadmium binding sites that are still available for up to 25 minutes testifies to a particular affinity between the electrode and this analyte. For the rest of the work, the value of 5 minutes was retained as the preconcentration time for the two metals.

#### 3.4.4. Study of the Effect of pH of Medium

**Figure 8:**
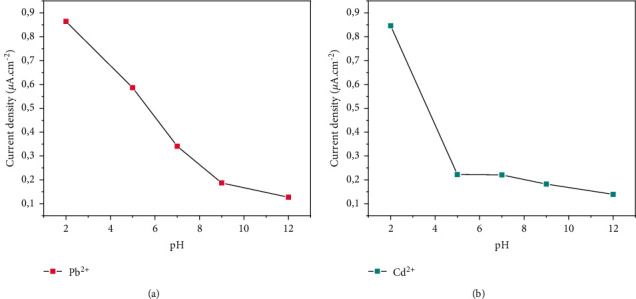
Effect of pH by SWV (Hg/Hg_2_Cl_2_; pH = 7; 0.1 M of Na_2_SO_4_).

In order to study the different forms of lead on the one hand and cadmium on the other hand in the study medium, the pH of the medium was varied from 2 to 12 as shown in Figures 8(a) and 8(b). This behavior was studied by square wave voltammetry, and the pH values were adjusted from 2 to 12. Figures 8(a) and 8(b) show that the current density is generally high in an acidic medium. This could be explained by the fact that, at low pH, metals are positively ionized, and therefore, the charge carriers are large enough to increase the current. As the pH increases, the current density drops significantly. The entry of metals into the hydrolysis mechanism could justify this attitude. Indeed, during the prolysis of water, there is production of hydroxide ions which can react with lead and cadmium to give the species Pb(OH)^+^ and Cd(OH)^+^. Under these conditions, the decrease in the available charges is accompanied by a drop in the current density at the surface of the electrodes. Under another prism, the adjustment of the pH of the reaction medium by soda (0.1 M) is likely to cause the formation of Cd(OH)^+^ and Pb(OH)^+^ cations which can continue to react to give the precipitates Cd(OH)_2_ and Pb(OH)_2_. Thereafter, the HCdO_2_^−^ and Pb(OH)_3_^−^ anions are formed. Since calcinated clay is predominantly made up of type 2 : 1 mineral, with an overall negatively charged surface, zero and negative charged hydroxides are disadvantaged in a process of electrostatic attraction. As for the anions formed, the electrostatic repulsion is an obstacle for their adsorption to the surface of the electrodes. Previous work [24–27] has shown similar results.

#### 3.4.5. Ionic Interference Effect

**Figure 9:**
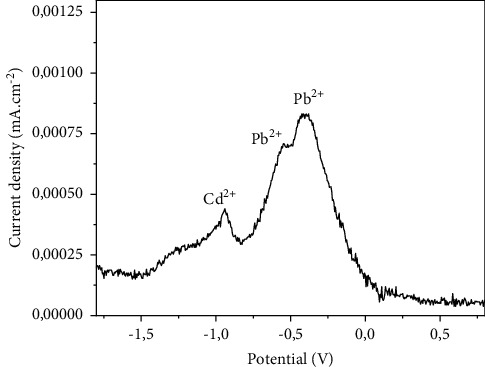
Ionic interference effect by SWV (Hg/Hg_2_Cl_2_; pH = 7; 0.1 M of Na_2_SO_4_).

In a medium containing both the metal ions Pb (II) and Cd (II), the ability of the proposed working electrode to simultaneously detect these metals was evaluated by SWV in 0.1 M Na_2_SO_4_. For this purpose, 1.2077 *μ*M of Pb^2+^ and 1.2966 *μ*M of Cd^2+^ were pooled, and the electrochemical response of the study is presented in Figure 9. The peak current equal to 0.441 mA/cm^2^ for a potential of −0.940 V reflects the oxidation of cadmium in its bivalent form. The shoulder between −0.550 V and −0.540 V whose peak current 0.709 mA/cm^2^ appearing exactly at −0.545 V corresponds to the reduction of metal ions Pb^2+^ to metal Pb. The most intense peak current (0.828 mA/cm^2^) obtained for a potential of 0.404 V corresponds to the oxidation of metallic lead to Pb^2+^ ion. These observations corroborate, on the one hand, the reversibility of the redox phenomenon with regard to lead and, on the other hand, the irreversibility of the phenomenon with respect to cadmium. According to the electrochemical response during the study of the interference effect, it is clear that the CPE-ACC electrode always remains selective for each metal.

### 3.5. Proposed Mechanism for Detecting Pb (II) and Cd (II)

**Figure 10:**
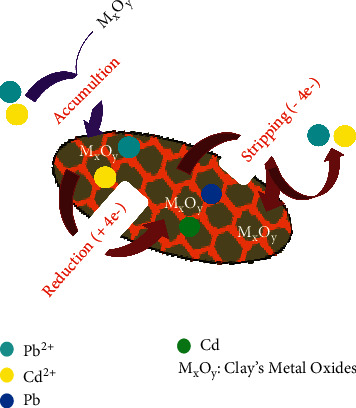
Schematic representation of detection mechanism for Pb^2+^ and Cd^2+^ ions.

The proposed mechanism for the simultaneous detection of Pb (II) and Cd (II) by SWV within the frame of this work could be based on several phenomena taking place in the reaction medium. Under a first prism, detection can be due to adsorption to the surface of the electrode by electrostatic attraction. Indeed, the clay consisting of metal oxides such as Al_2_O_3_, Fe_2_O_3_, SiO_2_, and CaO [28], heteroatoms (oxygen) can form coordination bonds with the electronic vacancies of Pb^2+^ and Cd^2+^. The graphite sheets have an interfoliar distance of around 0.335 nanometers [29]. The ionic radius of Pb (II) is of the order of 1.46 Å to 1.56 Å [30] and that of Cd (II) in the order of 0.95 Å [31], and an intercalation in the interfoliar spaces of clay and graphite can be considered during adsorption. From another angle, the ionic radius of Pb (II) being close to those of Fe (II) and Al (III), a replacement of the last one by the first one, can be considered during adsorption. After an open-circuit adsorption, the passage of current during the voltammetric analysis causes a reduction of the Pb^2+^ and Cd^2+^ ions each into its corresponding atom. The metals will then be stripped, and the ions will be released again into the electrolytic medium.  Figure 10 illustrates the mechanism of simultaneous detection.

### 3.6. Application of Electrodes to Tap Water Analysis

**Figure 11:**
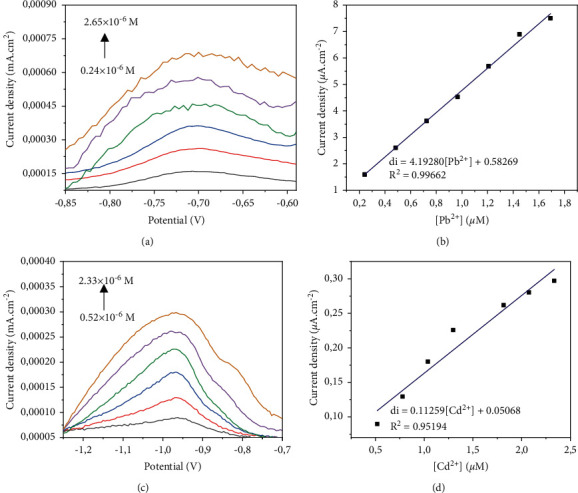
Effect of the concentration and calibration curve of the Pb^2+^ ions (a, b) and Cd^2+^ (c, d).

The CPE-ACC electrode was subjected to tap water for application in a real environment. The study was carried out by square wave voltammetry in a 0.1 M Na_2_SO_4_ solution. The lead (II) concentration was varied from 0.24 × 10^−6^M to 2.65 × 10^−6^M as shown in Figure 11(a). Regarding cadmium (II), the electrochemical responses were recorded in the concentration range 0.52 × 10^−6^M to 2.33 × 10^−6^M as shown in Figure 11(c). From the above, it is clear that the anode peak currents increase linearly with the Pb (II) concentration in solution with strong correlation (*R*^2^ = 0.99662, Figure 11(b)) for a detection limit equal to 0.08438 *μ*M. Moreover, the electroanalysis of Cd (II) also shows that the peak currents which evolve with the concentration of Cd (II) in the reaction medium. The correlation between peak current density and concentration is also high (*R*^2^ = 0.95194, Figure 11(d)) but not as much as that obtained with lead. The detection limit for the cadmium case was 0.46522 *μ*mol·L^−1^. These results show that the manufactured sensor detects lead (II) more reliably in distilled water than in tap water. With Cd^2+^ on the other hand, this sensor is more reliable in tap water than in distilled water during electrochemical detection.  Table 1 makes it possible to assess the reliability of the sensor according to the study environment and to compare it with other work.

## 4. Conclusion

The *in situ* detection of bivalent lead and cadmium was carried out by SWV via the graphite carbon paste electrode modified by acidified and calcinated clay from Makabaye (Far-North Cameroon). The modification of the graphite carbon paste electrode by an acid and calcination of the clay sample added value to the carbon electrode in terms of electrochemical recognition of divalent lead and cadmium metals. By subjecting the proposed working electrode to a medium containing both divalent lead and cadmium ions, the finding is that this electrode is capable of detecting these metal ions even in trace amounts. The detection is also perceptible in a real environment such as drinking water despite its complexity in terms of dissolved species. As a result, it can be said that the sensor developed is an electrochemical instrument that can be used for detecting the aforementioned metals *in situ*.

## Figures and Tables

**Table 1:** Comparison of the performance of some electrodes with other analytical methods for lead and cadmium.

Electrode material	Technique used	Solvent	Limit of detection		Reference
			Pb^2+^	Cd^2+^	
Ce-Zr/GCE	SWASV	Distilled water	5980 *μ*M	—	[5]
RGO-GNPs	SWASV	Distilled water	0.00058 *μ*M	0.00071 *μ*M	[6]
GCE-MWCNT/poly-PCV/Bi	DPASV	Distilled water	0.00193 *μ*M	0.00178 *μ*M	[8]
Fe_3_O_4_/MWCNTs/LSG/CS/GCE	SWASV	Distilled water	0.00034 *μ*M	0.00089 *μ*M	[21]
BiPs-CNFs/[EMIM][NTf2]/CPE	SWASV	Distilled water	0.00058 *μ*M	0.00222 *μ*M	[25]
ZnFe_2_O_4_-GCE	DPASV	Distilled water	0.00541 *μ*M	0.02224 *μ*M	[26]
GCE/ZSM-5/Pt	DPV	Distilled water	0.01196 *μ*M	—	[27]
CPE-ACC	SWV	Distilled water	0.15513 *μ*M	0.24227 *μ*M	This work
CPE-ACC	SWV	Tap water	0.08438 *μ*M	0.46522 *μ*M	This work

## Data Availability

No data were used to support this study.
